# Effectiveness of the level of personal relevance of visual autobiographical stimuli in the induction of positive emotions in young and older adults: pilot study protocol for a randomized controlled trial

**DOI:** 10.1186/s13063-020-04596-5

**Published:** 2020-07-20

**Authors:** Dolores Fernández, Laura Ros, Roberto Sánchez-Reolid, Jorge Javier Ricarte, José Miguel Latorre

**Affiliations:** 1 Department of Psychology, University of Castilla La Mancha, 02006 Albacete, Spain; 2 Computer Research Institute, University of Castilla La Mancha, 02071 Albacete, Spain; 3 IT Systems Department, University of Castilla La Mancha, 02071 Albacete, Spain

**Keywords:** Emotion regulation, Mood induction, Images, Autobiographical memories, Ageing, Personal relevance

## Abstract

**Background:**

The ability to retrieve specific memories is a cognitive and emotional protective factor. Among the most effective techniques to generate autobiographical memories is the use of audio-visual stimuli, particularly images. Developing and improving techniques that facilitate the generation of such memories could be highly effective in the prevention of depressive symptoms, especially in the elderly population. The aim of the present study is to examine how the level of personal relevance of pictures as autobiographical memory cues to induce positive emotions may affect an individual’s emotion regulation.

**Methods:**

The participants, 120 older adults aged 65 and over and 120 young adults aged between 18 and 35, of both sexes and without depressive symptoms, will be induced to a negative mood state by means of viewing a film clip. Following the negative mood induction, the participants will be shown positive images according to experimental group to which they were randomly assigned (high personal relevance: personal autobiographical photographs; medium personal relevance: pictures of favourite locations associated with specific positive autobiographical memories; and low personal relevance: positive images from the International Affective Picture System). We will analyse the differences in subjective (responses to questionnaires) and objectives measures (EEG signal, heart rate variability and electrodermal activity) between the groups before and after the induction of negative affect and following the recall of positive memories.

**Discussion:**

The use of images associated with specific positive autobiographical memories may be an effective input for inducing positive mood states, which has potentially important implications for their use as a cognitive behavioural technique to treat emotional disorders, such as depression, which are highly prevalent among older adults.

**Trial registration:**

ClinicalTrials.gov NCT04251104. Registered on 30 January 2020.

## Administrative information

Note: the numbers in curly brackets in this protocol refer to SPIRIT checklist item numbers. The order of the items has been modified to group similar items (see http://www.equator-network.org/reporting-guidelines/spirit-2013-statement-defining-standard-protocol-items-for-clinical-trials/).

**Table:** 

Title {1}	Effectiveness of the level of personal relevance of visual autobiographical stimuli in the induction of positive emotions in young and older adults: Pilot study protocol for a randomized controlled trial.
Trial registration {2a and 2b}.	ClinicalTrials.gov, NCT04251104. Registered on 30 January 2020.
Protocol version {3}	Issue Date: 20 December 2019 Protocol: Original Author(s): JML, LR and JJR
Funding {4}	This study will be supported by the Spanish Ministry of the Economy and Competitiveness/European Regional Development Fund under TIN2013-47074-C2-1-R and DPI2016-80894-R grants, and by the Castilla-La Mancha Department of Education, Culture and Sports and the European Regional Development Fund under SBPLY/19/180501/000181 grant.
Author details {5a}	Fernández, Dolores^1^, Ros, Laura^1^, Sánchez-Reolid, Roberto^2,3^, Ricarte, Jorge Javier^1^ and Latorre, José Miguel^1^ ^1^Department of Psychology, University of Castilla La Mancha. 02006-Albacete, Spain. ^2^Computer Research Institute, University of Castilla La Mancha. 02071-Albacete, Spain. ^3^IT Systems Department, University of Castilla La Mancha. 02071-Albacete, Spain
Name and contact information for the trial sponsor {5b}	Trial Sponsor: University of Castilla La Mancha (Spain) Contact name: Antonio Fernández Caballero Address: High Technique School of Industrial Engineers. Avda. España s/n, 02071-Albacete (Spain) Telephone: +34 967 59 92 00 Ext. 2406 E-mail: antonio.fdez@uclm.es
Role of sponsor {5c}	The funding source had no role in the design of the study and will not have any role during its execution, analyses, interpretation of the data, or decision to submit results.
Organizational structure and responsibilities {5d}	JML and LR are the leading investigators. They are the main persons responsible for the design and conduct of the study and for the budget administration. Responsibilities common to all researchers (JML, LR, JJR, RSR, and DF) are listed below: - Agreement of final protocol - Study planning - Reviewing progress of the study Additionally, JML will be responsible for organizing recruitment of participants. JJR and DF will organize the screening and experimental phases. LR and RSR will be responsible for the data management. Periodic meetings of all researchers will be established every 15 days to review and evaluate the progress of the study. Stakeholders will be represented by the management team of the Alumni Association of the University of Experience (ALUEX). They will meet periodically every 15 days with the leading investigators during the data collection phase. These meetings are intended to provide feedback to the research group about positive and/or negative assessments of the individuals’ participation in the study. This feedback will be used to assess a possible modification of the protocol if necessary. Any protocol modification will require formal approval from the Clinical Research Ethics Committee of the Castilla-La Mancha Health Service. Following the completion of data collection, bi-monthly meetings will be held to inform the stakeholders of the progress and results of the trial until the end of the study.

## Introduction

### Background and rationale {6a}

The probability of suffering from depression at some point in one’s life is superior to that of other mental disorders, such as anxiety, for example [1]. Its prevalence is elevated, and it is estimated that between 10 and 20% of the population will develop a depressive disorder during their lives [2]. Depression can thus be considered one of the most common health disorders, especially in older adults, given that clinically significant depressive symptoms are present in 15% of community-dwelling older adults [3] and in 46.5% of institutionalized older persons [4]. Depressive symptoms in older adults may be the result of multiple changes occurring in this life period (e.g. loss of loved ones, worsening health, social isolation, reduced independence, etc.). A number of authors have suggested that when older adults are unable to cope with and adapt to these new circumstances, depressive disorders may appear [5]. Furthermore, depressive symptomatology in older people is associated with a higher risk of morbidity, self-neglect, suicide, impaired physical, social and cognitive functioning, decreased quality of life and even a higher risk of mortality [3, 6, 7]. Consequently, preventing and mitigating depressive symptoms should be a core element in improving mental health and quality of life in healthy older adults. For this reason, studies should not be directed solely towards the development and improvement of treatments for clinical depression, but also towards studying variables that can help prevent the appearance of depressive symptoms and/or reduce the presence of non-clinical depressive symptoms in healthy ageing.

#### Emotion regulation in ageing and depressive symptomatology

Depressive symptomatology represents a significant shift away from the affective balance within which a person moves, and as such, they are related to problems or difficulties in emotion regulation. Hence, successful emotion regulation may increase positive affect and diminish negative affect [8].

How individuals manage emotions, be they positive or negative, changes as we grow older, and it has been suggested that, despite the impairment of cognitive functioning in ageing, emotion regulation may improve with age [9]. In the same line, studies have reported a change in priorities with age, with older adults tending to focus on positive emotional goals, distancing themselves from negative information or mood states, generating, in turn, a positivity effect, whereby the recall of positive information increases significantly [10], and negative situations are avoided [11]. An example of this positivity effect is found in autobiographical memory recall, where the number of positive memories retrieved tends to increase with age [12], since, when recalling past events, older adults typically remember them more positively compared to young adults [13, 14]. Nonetheless, some studies have suggested an opposite effect, where the use of emotion regulation strategies reduces with age [15]. Finally, some authors have even posited that emotion regulation is unaffected by ageing [16, 17].

#### Autobiographical memory for positive mood induction

Over the years, different effective emotion regulation techniques have emerged, with one of the most significant being the use of autobiographical memories. These techniques are based on the idea that individuals are able to regulate negative emotional mood states using personal episodic information stored in memory [18]. Various studies have demonstrated that training access to specific positive events (referring to something with positive emotional content that occurred at a concrete moment in a specific space and time, lasting from seconds to hours) is effective in regulating emotions and improving mood state in older adults, reducing negative mood and increasing positive emotions [19]. Taking these results into account, cognitive interventions aimed at increasing specific positive autobiographical memories (e.g. life review [20] or memory specificity training (MEST) [21]) have been developed. These therapies have been shown to be a successful method of reducing depression through improving autobiographical memory specificity [22]. This mood improvement is even possible after the induction of a negative mood state [23, 24]. Additionally, a number of studies have also reported that the ability to retrieve specific autobiographical memories is an indicator of emotional well-being [25] and is correlated with enhanced life satisfaction [26].

One of the key types of autobiographical memories is self-defining memories. Characterized by the vividness and intensity of their affective tone and related to self-discovery, self-understanding and self-images, these memories help us to create and understand our life story [27]. Integrating such memories to enrich our self-understanding is a predictor of more successful emotion regulation [28]. In older adults, self-defining memories are associated with personal maturity, emotion regulation and the creation of meaning [29]. Compared with younger adults, older people’s self-defining memories present greater levels of vividness, importance and positive affect, especially with regard to significant relationships, achievements, bringing up children, etc., although their specificity is lower compared to younger adults’ memories [29].

Autobiographical memories have been used as a procedure for inducing different mood states, being especially effective in the induction of positive mood states [30, 31]. These procedures based on autobiographical memory recall require participants to generate the emotion they felt when they experienced a specific personal event, recalling it as vividly as possible and re-experiencing sensations, emotions, perceptions and reactions [32]. El-Ziab posits that the more accessible and meaningful the autobiographical memories are for the participant, the more effective these procedures are in regulating negative mood [33].

#### Importance of the use of images in mood induction

As suggested by Holmes and Mathews [34], negative mood state is typically accompanied by distressing mental images. In this sense, several studies have shown that imaging positive events enhances mood more than verbal processing [35, 36]. These findings have motivated the development of techniques based on visual stimuli to reduce these types of symptoms and generate positive emotions [37]. According to Sitaram et al. [38], pictures have proven to be one of the most effective cues for capturing the complexity of an emotional recall scenario. Their effectiveness might lie in the fact that, compared to other types of stimuli, images have a direct correspondence to sensory experience and are thus more real [39], so helping a person to access emotions related to their autobiographical memories [40].

Given the importance of autobiographical memory and images when inducing positive mood states, it seems necessary to determine what types of pictures might be more effective in eliciting mood states through the retrieval of memories related to specific positive events. A number of studies have reported that viewing standardized images, such as those taken from the International Affective Picture System (IAPS) [41], can activate motives, albeit less strongly than life’s real transactions, given that the personal relevance or ecological validity for the participant of such images may be low [42]. This finding suggests hypotheses related to the possibility that images with greater personal relevance for a participant, that is, an individual’s own photos, might significantly increase the induction of a positive emotional state.

#### Objective measurement of emotional response: physiological correlates

Among the methods most widely used to objectively assess emotional response are brain activity measurement (e*lectroencephalogram*) and *physiological measures*, such as *electrodermal activity* and *heart rate*.

##### Measurement of brain activity

Emotions generate temporal patterns of brain activation [43], which can be identified and discriminated using the *electroencephalogram* (EEG) [44, 45].

The use of the EEG signal has facilitated the study of the neurophysiological correlates through the amplitude and frequency changes generated in response to different stimuli (e.g. picture viewing) [46], enabling different emotional states to be distinguished. A number of studies have reported that low frequency bands are associated with emotional processing [47, 48] and, alpha bands, specifically, are related to positive emotions [49]. On the other hand, high frequencies (gamma bands) are relevant to cognitive processes that include feelings and emotions [50]. According to Li and Lu [51], these bands are especially significant for the classification of emotions using images as stimuli.

Other studies have shown that the left and right frontal regions are associated with positive and negative emotional responses, respectively [52]. In the same line, research has mainly focused on analysing alpha band asymmetries in the frontal cortex to understand the hemispheric specialization of emotions [53]. Findings suggest that, compared with negative emotions, positive emotions present higher frontal coherence as regards the alpha and beta bands in the right superior parietal lobe [54]. Other studies have reported decreased alpha and theta power in the left frontal region in response to positive processing, and a decrease in the same bands in the right frontal region triggered by negative processing, which suggests band power is associated with the valence of the stimulus [55].

Marosi et al. [56] found that higher delta frequencies in left and right frontal, parietal and temporal regions and high beta frequencies in the left fronto-temporal and temporal regions are associated with joy. In addition, higher delta and alpha frequencies appear in response to exposure to pleasant images [46]. These findings corroborate previous studies finding greater alpha band activity in the left frontal region during positive emotion processing [57]. Moreover, it has been shown that high arousal stimuli generate a decline in alpha power [58] and increased delta and theta power [53, 59]. Studies have also revealed that positive stimuli, such as pleasant odours, happy musical excerpts and pleasant TV commercials, may induce significantly lower frontal alpha power and higher theta power in the left hemisphere [60–62]. Strong synchronization has been found between theta and delta waves in response to emotional stimuli [48], which suggests that synchronization between these two brain waves during autobiographical memory tasks may be considered a marker of emotional arousal [63].

Autobiographical memory has been associated with a network of predominantly lateralized brain regions, and thus, mood induction procedures using autobiographical memory tasks generate bilateral activation [64]. Specifically, a pattern of left lateralization during the initial search for general autobiographical knowledge has been found, while the subsequent re-experiencing of the memory has been associated with the right hemisphere, especially the posterior cortical regions [65], also preferentially associated with emotional processing and social cognitive processes [66, 67].

##### Autonomic nervous system

As previously mentioned, emotions involve psychophysiological processes, and thus generate changes in variables such as heart rate and blood pressure [68] or galvanic skin response [69].

A number of studies have reported higher heart rate variability (HRV) in older adults as self-reported positive emotion and well-being increase [70], with high HRV being associated with greater emotional well-being and better emotion regulation [71–73]. Then, the health benefits of positive emotions on health may derive from reduced cardiovascular activation associated with decreased sympathetic nervous system (SNS) activation and an increase in activation of the parasympathetic nervous system (PNS) [74]. Furthermore, positive emotions facilitate cardiovascular recovery following negative emotions [75]. Low heart rate has been associated with pleasant or relaxed emotional states, while heart rate accelerations occur in response to intense emotional states, sexual arousal or mental effort [76].

Furthermore, electrodermal activity (EDA), or galvanic skin response (GSR), has been associated with SNS activity [77, 78]. Studies such as that by Codispoti et al. reported higher skin conductance response to exposure to emotional pictures [79]. The use of EDA is highly effective in measuring the level of an individual’s emotional arousal, regardless of the emotional valence of the stimuli, since it is able to quantify changes in the SNS [80]. Various studies have identified changes in EDA (increase in peaks) following exposure to images taken from the IAPS [81].

## Objectives {7}

The ability to retrieve specific memories is a cognitive and emotional protective factor. Thus, developing and improving techniques that facilitate the generation of such memories, which are able to enhance mood state, could be highly effective in the prevention and/or reduction of depressive symptoms in healthy older adults. Consequently, the aim of the present study is to delve deeper into the knowledge and effectiveness of different visual stimuli in inducing positive emotions and reducing negative emotions, examining how the level of personal relevance of pictures as autobiographical memory cues may affect an individual’s emotion regulation. Importantly, this new knowledge could also be used in interventions focused on the training of specific positive memories with the aim of improving its efficacy in the treatment of clinical depression in the elderly population.

Additionally, given the differences in emotion regulation between young and older adults, we aim to assess whether the influence on emotion regulation of the level of personal relevance of the images differs according to a person’s age.

The main aim of this study, then, is to experimentally evaluate how the level of personal relevance of the images used as stimuli to access specific positive autobiographical memories can improve emotion regulation, especially in older adults. From this primary aim, we derive the following specific objectives:

1. To experimentally study the impact of the personal relevance of pictures on the improvement of mood state, following negative mood induction. To this end, we will use three types of images, classified according to their personal relevance: (a) personal autobiographical photographs (high personal relevance), (b) images of locations related to the participants’ lives (medium personal relevance) and (c) images from the IAPS (low personal relevance).
  Additionally, as suggested by Talarico et al. [82], events remembered with a high sense of reliving induce stronger emotions. For this reason, we will also examine the degree of reliving in the autobiographical memories evoked by the three types of images.
2. To analyse any age-related differences in the effectiveness of the use of the three types of images to regulate emotion. We will compare the efficacy of the three categories of pictures (high, medium and low relevance) in inducing positive mood states in a group of young adults and a group of older adults.

The objective measures of emotion (EEG, EDA and HRV) will be used to substantiate the effects of emotion induction mechanisms observed in self-evaluations.

## Trial design {8}

This is an exploratory randomized controlled study designed to compare the effectiveness of three types of autobiographical stimuli, classified according to their level of personal relevance, in the induction of positive emotions resulting from the retrieval of specific positive autobiographical memories. The study sample will comprise a group of adults aged 65 years and over and a group of young adults aged between 18 and 35. Each participant will be randomly assigned to one of three parallel groups (high, medium and low personal relevance images). The allocation ratio will be 1:1:1.

## Methods: participants, interventions and outcomes

### Study setting {9}

The study sample will comprise a group of healthy adults aged 65 years or over and a group of healthy young adults aged between 18 and 35. The first group will consist of adults aged 65 years or over, recruited in the city of Albacete (Spain) from senior citizens’ associations and centres, from a cultural association for older people (Alumni Association of the University of Experience [*Asociación de Alumnos de la Universidad de la Experiencia*, ALUEX]), from the José Saramago University Programme for older adults and from different socio-cultural centres. The second group will comprise young adults, aged between 18 and 35 years, who will be recruited from among students and workers at the University of Castilla-La Mancha (Albacete Campus), Spain.

The study will include both men and women, aiming for a proportion of at least 40% and 60%.

#### Eligibility criteria {10}

All participants interested in the study will be asked to complete preliminary questionnaires to assess their eligibility before being assigned to the experimental groups.

Participation will be dependent on the following inclusion criteria:

1. Older adults will present no symptoms of cognitive impairment. The self-administered Test Your Memory (TYM) will be used to assess cognitive performance [83, 84].
2. Absence of depressive symptomatology, which will be assessed using the Patient-Reported Outcomes Measurement Information System-Depression [85].
3. Given the high comorbidity between anxiety and depression [86, 87], it was decided that both young and older participants should present no symptoms of anxiety. This will be assessed using the Patient-Reported Outcomes Measurement Information System-Anxiety [85].
4. No sensory deficits that might impact performance in the experiment and the psychological tests.
5. Sufficient literacy skills to understand the instructions for the experiment and the psychological tests.
6. Signed informed consent.

#### Who will take informed consent? {26a}

Two trained research psychologists will present the trial to possible participants. Participants will then receive information sheets and any questions about the study will be resolved. Finally, research psychologists will obtain written consent from those willing to participate in the trial.

#### Additional consent provisions for collection and use of participant data and biological specimens {26b}

Not applicable. Participant data will not be used for purposes that are separate from the main trial.

### Interventions

#### Explanation for the choice of comparators {6b}

The conditions of medium and high personal relevant images will be compared with IAPS images (low personal relevant condition). IAPS [41] is traditionally considered a valid system for affectively eliciting stimuli that can be used as a representative tool, for example, in mood and emotion induction studies. For this reason, its selection as comparator is justified.

#### Intervention description {11a}

##### Screening phase

The first screening phase will be conducted with all the potential participants, both young and older adults. The screening phase will take place in different sessions in the different recruitment centres until the minimum necessary number of participants is achieved. The sessions will be conducted in both group and individual formats. The instruments described in the corresponding section will be administered and individuals not meeting the inclusion criteria will be excluded from the study.

##### Pre-experimental phase

**Fig. 1:**
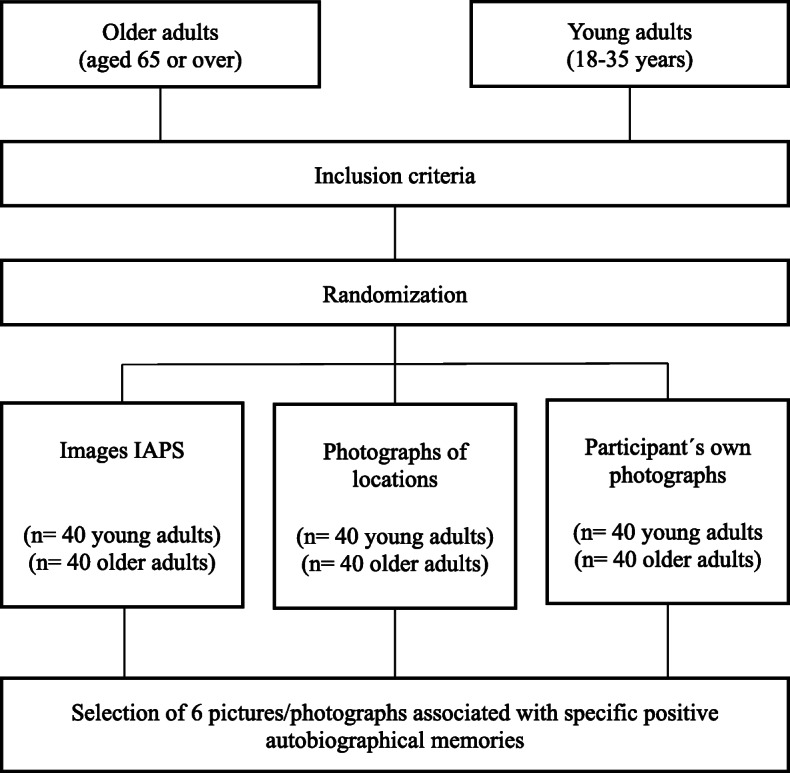
Randomization phase

The participants from both groups (older adults and young adults) will be randomly assigned to one of the three experimental groups, the condition of which will differ according to the type of stimulus used to elicit specific positive autobiographical memories: (1) low personal relevance condition: positive images taken from the International Affective Picture System [41]; (2) medium personal relevance condition: pictures of places related to the participants’ lives (the most important or characteristic places in their town or city, or a place they have visited, etc.); and (3) high personal relevance condition: participants’ own photographs related to their positive autobiographical experiences (see Fig. 1).

We will thus have three types of images varying in their level of personal relevance for each participant. The first type of images is pictures taken for the standardized IAPS. There will be a total of 40 pictures selected by the research team according to their level of positive valence (emotional valence values between 7.03 and 8.34). The participants will be asked to choose the 6 photos that best enable them to generate a specific positive autobiographical memory.

The second type of images used will be photos of locations and places that are significant for the participant, which will be selected following a prior interview in which the participant will be asked to name places that evoke specific positive autobiographical memories. These images will be taken from the Internet. We will select various images of each location, giving participants the opportunity to choose the one which best fits their memory.

The third type of images will be autobiographical photographs selected by participants from their own collection. Participants will be asked to previously select six photos, for which they can recall the exact moment they were taken and which are related to events that have made them feel happy during their lives. These six events will be those they are asked to recall in the experimental task.

For each of the memories retrieved in the three conditions, the following factors will be assessed: (1) the degree of nostalgia generated by the memory; (2) the intensity of the positive emotion evoked by the memory; (3) whether the participant was a spectator in the event (“as if I were watching a film”) or an actor (“as if I were seeing it with my own eyes or recording it”); (4) the significance of the event in the person’s life; (5) personal growth since the event occurred; (6) belief that the event has led them to better understand themselves and what life means; and (7) belief that they have learnt more about what life means as a result of the event.

##### Experimental phase

**Fig. 2:**
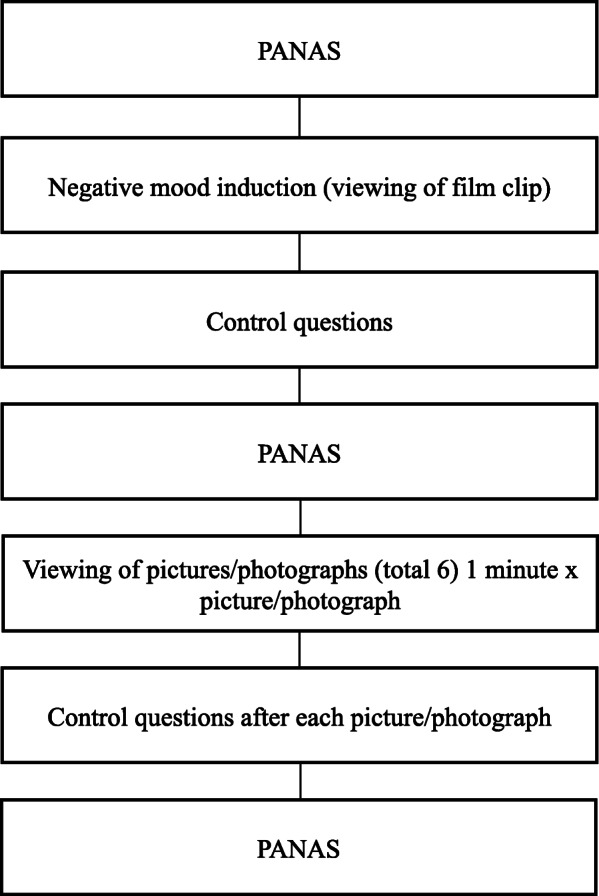
Experimental phase

The experimental phase (see Fig. 2) comprises the three previously described experimental conditions. During the experimental task, the participants will be wearing the devices described in the instruments section to measure electrophysiological signals. The experiment will be conducted in a controlled environment, which will be comfortable and noiseless, and where the temperature and humidity in the room will be controlled. The researcher will leave the room at the beginning of the experiment to avoid any possible conditioning.

At the start of the experiment, all the participants will be asked to complete the PANAS mood state scale [88]. This scale will be completed on three occasions: at the start of the task, following the negative mood induction (viewing the film clip) and after the autobiographical memory task (presentation of images according to the assigned experimental condition).

After completing the PANAS [88], the negative mood induction phase will begin. The participants will watch a 7-min section of the film “Dead Man Walking” (Polygram Filmed Entertainment, Havoc, Working Title Films), which shows the execution of a prisoner sentenced to death. Before seeing the film clip, the participants will be encouraged to experience the feelings generated by the clip as intensely as possible. After viewing the film clip, they will be asked to answer the control questions related to their level of concentration while watching the clip, and the degree to which they surrendered to the feelings it generated. They will then be asked to complete the PANAS [88] again in order to assess the effectiveness of the negative mood induction procedure.

Following the negative mood induction phase, the emotional recovery phase will be initiated, in which the participants will look at a total of six pictures previously selected according to their experimental condition in order to generate specific positive autobiographical memories. Each image will be presented for 1 min, and during this time, the participants will be asked to concentrate as hard as they can on the memory that each picture evokes with the aim of reliving the positive emotions they felt at that moment as intensely as possible. After each picture, the participants will answer a series of control questions related to their level of concentration while looking at the pictures and to what extent they were able to relive the emotions they felt at the moment related to the images. At the end of this phase, participants will once more complete the PANAS [88] in order to assess the effectiveness of the emotional recovery.

#### Criteria for discontinuing or modifying allocated interventions {11b}

Not applicable. The interventions will not be modified. Participants may leave the trial at any time if they wish. Participants who do not complete the entire trial will be excluded from the study.

#### Strategies to improve adherence to interventions {11c}

Not applicable. The experimental intervention is performed in a single session, so it is not necessary to develop techniques to improve adherence to interventions.

#### Relevant concomitant care permitted or prohibited during the trial {11d}

Not applicable. The experimental intervention is performed in a single session, so it is not possible for the participant to receive concomitant cares during the trial.

#### Provisions for post-trial care {30}

No adverse effects are expected after the experimental session. However, if after the phase of negative emotional induction, the intervention aimed at their emotional recovery was not effective, the psychologist responsible for the session, using the appropriate psychological techniques, will be responsible for the individual having recovered the emotional state previous to the start of the experimental session.

### Outcomes {12}

Participants will first complete a sociodemographic questionnaire to collect data on the following variables: age, sex, educational level, current occupation, marital status relationship status, health problems, regular medication and self-perceived health status. Additionally, participants will be asked to complete a series of screening questionnaires.

#### Screening measures

##### Test Your Memory (TYM [83], Spanish adaptation [84])

The TYM is a cognitive screening test to detect Alzheimer’s disease and mild cognitive impairment. It consists of a set of 10 tasks, with a possible total score of 50 points. The following abilities are measured: orientation, ability to copy a sentence, semantic information, calculation, verbal fluency, similarities, confrontation naming and perception. The help required is scored on a 0–5 range, where 5 is no assistance at all. The higher the score obtained, the better is the performance. The cut off for cognitive impairment is 40 or below and 36 or below for dementia. The Spanish version has demonstrated adequate psychometric properties (*α* = .86) [84].

##### Patient-Reported Outcomes Measurement Information System-D (PROMIS®-Depression 4ª, short form) [85]

This version includes four items to measure a participant’s negative affect in the past 7 days, with five response options ranging from 1 = never, to 5 = always, where the higher the score, the greater is the negative affect. The test forms part of PROMIS-29, and total scores range between 4 and 20, where the highest scores correspond to more serious depressive symptoms [89]. The cut off is set at 11 points (http://www.helathmeasures.net). Indices of reliability for this version are excellent (*α* = .96).

##### Patient-Reported Outcomes Measurement Information System-A (PROMIS®-Anxiety 4a, short form) [85]

This version includes four items to measure symptoms of anxiety in the past 7 days with five response options ranging from 1 = never, to 5 = always, where the higher the score, the higher is the anxiety. It forms part of PROMIS-29, and total scores range between 4 and 20, where the highest scores correspond to the most severe symptoms of anxiety [89]. The cut off is set at 11 points (http://www.healthmeasures.net). Indices of reliability for this version are excellent (*α* = .96).

#### Mood state measures

##### Positive and Negative Affect Schedule (PANAS) [88]

This scale is composed of two 10-item subscales that measure the primary dimensions of mood state (positive affect and negative affect). The items describe adjectives associated with common feelings and emotions and are scored on a 5-point Likert-type scale ranging from 1 (very slightly or not at all) to 5 (extremely). The test provides separate scores for the two types of affect, and an affective balance score can be obtained by subtracting the negative affect score from that for positive affect [90], meaning that the higher the score, the greater is the predominance of positive affect. The validated version for Spanish population [91] has obtained alpha coefficients between .87 and .91.

#### Measurement of emotional experience in the experiments

##### Self-Assessment Manikin (SAM) [92]

This test measures five sub-dimensions (5 figures per dimension): (a) affective valence, pleasure or hedonism (SAM-Val), with manikins that range from smiling and happy to frowning and unhappy; (b) arousal or activation (SAM-Act), ranging from a wide-eyed figure to a sleepy one; and (c) dominance or controlled emotion (SAM-Dom), where the figures range from a small manikin indicating minimum control to a large, impassive figure. As the instrument requires no use of language, it is free of cultural influences and is thus adequate for use in different countries and cultures.

#### Control questions

##### Control questions following negative mood induction

To measure the participant’s level of immersion in the film clip used for negative mood induction, the following questions are used: (1) On a scale from 0 to 9, indicate your level of concentration while watching the film clip (0 = no concentration; 9 = total concentration), and (2) on a scale from 0 to 9, indicate to what degree you surrendered to the feelings generated by the film clip (0 = I did not surrender at all to the feelings in the film clip; 9 = I surrendered totally to the feelings in the film clip).

##### Control questions following exposure to the pictures/photographs

To assess the extent to which the participant felt involved in viewing the images chosen to elicit positive affect, the following questions are included: (1) On a scale from 0 to 9, indicate your level of concentration when recalling the event (0 = total lack of concentration; 9 = total concentration), and (2) on a scale from 0 to 9, indicate to what degree you were able to relive the positive emotions you felt at the moment of the event (0 = I was unable to relive the emotions; 9 = I was able to relive the emotions completely).

#### Devices used to acquire physiological signals and behavioural data

##### Brain Computer Interface (BCI)

BCI systems are able to detect, process and respond to affective states by means of physiological signals [93] and the analysis of frequency bands associated with such emotional states. EEG data will be obtained wirelessly using the EMOTIV Epoc+ (http://www.emotiv.com/epoc), which is formed by 14 electrodes, divided into sensors and two reference electrodes placed across both hemispheres. The electrodes are labelled according to the International 10-120 positioning system (AF3, F7, F3, FC5, T7, P7, O1, O2, P8, T8, FC6, F4, F8, AF4 and the reference electrodes P3 and P4).

##### Empatica E4

This device records heart rate and electrodermal activity signals (http://www.empatica.com/en-eu/research/e4/). It is a wireless device able to measure variables such as heart rate variability, interbeat interval, electrodermal activity, acceleration and skin temperature (HRV, IBI, EDA, ACC, TMP, respectively). It is one of the most efficient devices in the field and is certified by the US Food and Drugs Administration for medical use.

##### Software E-PRIME 3.0

This system allows psychological experiments to be designed, generated and reproduced and also records the experimental data and enables the data to be edited and analysed. It can play films and videos, digitally record participants’ sonic responses and can also be integrated with other equipment, such as EEG devices. It will be used to design the experimental task and collect and analyse the subjective data.

### Participant timeline {13}

Participant recruitment is expected to begin in October 2020 and continue for 2 months. Nevertheless, if the minimum number of participants in each experimental group is not reached in early December, the recruitment period will be extended until the required minimum is reached. In this phase, those who agree to participate will complete the screening questionnaires.

The pre-experimental and experimental phases will take place 7–21 days after randomization. Both phases will take place on the same day. In the pre-experimental phase, participants will select the images to be displayed in the experimental task according to the group to which they are assigned (low, medium or high personal relevance). Finally, in the experimental phase, they will complete the mood questionnaires and the experimental task.

**Table:** 

Activity/assessment	Approximate time to complete	TIME			
		− 1	0	1	
		Screening phase	Randomization	Pre-experimental phase	Experimental phase
Informed consent	5 min	X			
Test Your Memory	5 min	X			
PROMIS-Depression	5 min	X			
PROMIS-Anxiety	5 min	X			
Demographic questionnaire	10 min	X			
Assessment of inclusion criteria		N/A	X		
Randomization		N/A	X		
Selection of images for emotional recovery	25 min			X	
PANAS pre-emotional induction	5 min				X
Experimental task and questions about emotional experience during the task	25 min				X
PANAS post-emotional induction	5 min				X
PANAS post-emotional recovery	5 min				X

### Sample size {14}

This study requires a sample of 120 participants aged 65 or older (divided into three groups of 40) without cognitive impairment or major depressive disorder, and 120 young adults aged between 18 and 35 (divided into three groups of 40) with no major depressive disorder.

To calculate the sample size, we used GPower version 3.1., considering the following parameters: a 95% confidence interval (*p* < .05), an estimated power of 95%, a predicted medium effect size of 0.06 (partial eta squared; Cohen [94]), 6 repeated measures (6 pictures), an expected correlation between of .80 and 6 experimental groups (3 types of pictures × 2 age groups).

### Recruitment {15}

People interested in participating in this study will be provided with a detailed information sheet supplemented with a verbal explanation of the study procedure. If the participants agree with this information, the screening questionnaires will be completed. The research psychologists will obtain the informed written consent of each participant prior to the screening phase. The questionnaires in this phase will include a complete sociodemographic history, a screening test to assess the presence of cognitive impairment and scales to measure anxiety and depression symptoms. Of these individuals, all participants meeting the inclusion criteria will be recruited for the study. Recruitment will continue until the minimum number of participants in each group is reached.

### Assignment of interventions: allocation

#### Sequence generation {16a}

The online software Randomizer version 3.0 [95] will be used to randomly assign 40 participants to each of the three experimental conditions within each group (young and older adults). The random assignment will be conducted by a third party in order to ensure the researcher remains blind to the study.

#### Concealment mechanism {16b}

Participants will be randomly assigned to one of three groups (high, medium and low personal relevance images) with a 1:1:1 allocation as per the online software Randomizer version 3.0 [95]. Allocation concealment will be ensured as the randomization code will not be released until the participant has been recruited into the study, which takes place after all baseline measurements have been completed and after verifying that the inclusion criteria for participation are met and that informed consent has been provided.

#### Implementation {16c}

All patients who give consent for participation and meet the inclusion criteria will be randomized.

The random assignment of participants will be conducted by a member of the Psychology Department blinded to the objectives of the study. This staff member will send a third research psychologist a list with the random assignation of each participant to each intervention group. This psychologist will not be involved in the recruitment of participants or in assessing the outcomes of the study. The psychologists responsible for participant recruitment will not be allowed to receive information about group allocation.

Throughout the study, the randomization will be conducted by the same member of the Psychology Department in order to keep the data management and the statistician blind to the study condition. Thus, randomization will be conducted without any influence from the researchers involved in the study.

### Assignment of interventions: blinding

#### Who will be blinded {17a}

The psychologists responsible for participant recruitment will be blinded to the information about group allocation. Due to the nature of the intervention, neither participants nor psychologists responsible for the experimental phase can be blinded for the allocation, but the psychologist will be trained to reduce her influence on the participants as much as possible. To this end, she will be provided with clear steps and norms to follow during the experimental intervention.

#### Procedure for unblinding if needed {17b}

Unblinding should not be necessary in this study.

## Data collection and management

### Plans for assessment and collection of outcomes {18a}

Each psychologist participating in the data collection will be trained in the study requirements and the steps and specific norms to follow while the questionnaires and experimental tasks are administered.

A detailed description of the study instruments and their validity can be found in the outcomes section. The informed consent form and sociodemographic questionnaire are included in Additional files 1 and 2, respectively.

### Plans to promote participant retention and complete follow-up {18b}

Not applicable. The experimental intervention is performed in a single session, so it is not necessary to develop techniques to promote participant retention and complete follow-up.

### Data management {19}

Data from the screening phase will be entered and kept in a SPSS file by the research psychologists responsible for the data collection in this phase.

In the experimental phase, subjective questionnaires will be answered using e-prime software. This data will be added to another SPSS file. The same procedure will be conducted with the objective measures (heart rate variability, electrodermal activity and EEG), once these signals have been processed. All participants will receive the same identification in all SPSS files (screening, subjective measures and objective measures).

The psychologists responsible for the data analysis will merge the SPSS files to create a single SPSS file including all the data.

Participant files will be stored in numerical order in a secure and accessible place and manner. These files will be held in storage for a period of 3 years after completion of the study.

### Confidentiality {27}

All the study-related information will be stored securely at the study site. All participant information will be stored in locked file cabinets in areas with limited access. All questionnaire forms will be identified only by a coded ID (identification) number in order to maintain participant confidentiality. All records containing names or other personal identifiers will be stored separately from study records identified by a code number. All databases will be secured with password-protected access systems. Lists that link participant ID numbers to other identifying information will be stored in a separate, locked file in an area with limited access.

Participants’ study information will not be released outside the study without their written permission.

### Plans for collection, laboratory evaluation and storage of biological specimens for genetic or molecular analysis in this trial/future use {33}

Not applicable. This trial does not use biological specimens.

## Statistical methods

### Statistical methods for primary and secondary outcomes {20a}

#### Statistical analysis of the subjective variables

All analyses will be conducted using SPSS24.0 software. Firstly, ANOVAs and chi-square tests will be conducted to determine whether significant differences exist between the different intervention groups for the variables collected (PROMIS and PANAS scales) in the first assessment phase (Time 1) and for the level of immersion when viewing the film clip during the negative mood induction. If differences between groups are found in any of these variables, the variables with statistically significant differences will be included as covariate variables in the following ANOVAs. Secondly, to assess the impact of personal relevance of the images in emotional recovery, a repeated-measures ANOVA and post hoc Bonferroni tests will be performed in a mixed design with type of image (low, medium and high personal relevance groups) and group (young and older) as between-subjects variables and time (pre-test—Time 1—, post-negative emotional induction—Time 2—and post-positive emotional induction—Time 3) as within-subject variables. Finally, to assess the sense of reliving for the three groups of images, a repeated-measures ANOVA and post hoc Bonferroni tests will be performed in a mixed design with type of image (low, medium and high personal relevance groups) and group (young and older) as between-subjects variables and (average score of concentration level recalling the events and average score of the degree of reliving the positive emotions of the remembered events) as within-subject variables.

#### Processing and statistical analysis of objective variables

##### Heart rate variability (HRV) and electrodermal activity (EDA)

- Data processing

Cardiovascular and electrodermal activity variables will be measured through blood volume pressure (BVP) and skin conductance, respectively, using the Empatica E4 device. Alternations of the BVP waveform are highly correlated with heart ventricular depolarization and repolarization, thus being suitable to measure heart rhythm [96]. BVP and SC will be recorded at a sampling rate of 100 Hz and 4 Hz, respectively. Once data are acquired, BVP and SC signals will be processed to reduce noise. To avoid noise, different filters will be applied to both signals. Specifically, for the BVP signal, baseline drift will be removed by carrying out a 0.5-Hz cut-off-high-pass, linear phase finite impulse response (FIR) filter. Then, a 30-Hz cut-off low-pass, linear-phase FIR filter will be used to eliminate high-frequency noise and power-line interferences. Additionally, peaks related to pulse pumping will be located on BVP signals using a robust and reliable peak detection algorithm, capable of dealing with movement artefacts and signal morphologies [97]. Finally, interbeat intervals (IBIs) will be derived from the peak-data series.

Related to the SC signal, SC morphology is the result of two independent components: a fast-changing skin conductance response (SCR), overlapped with a slowly changing skin conductance level component (SCL). The SCL component ranges from 0 to 0.05 Hz, while SCR ranges from 0.05 to 1.5 Hz. Each SC signal will be filtered by applying a 1.5-Hz cut-off low-pass FIR filter to decrease noise generated during the acquisition.

Regarding heart rhythm, the IBI data series will be transformed to obtain the heart rate (HR) measured in beats per minute (BPM). Then, the HR metric will be partitioned into 5-s equally separated segments. Finally, the mean HR will be stored and used subsequently in the statistical analysis. A similar procedure will be carried out with the SC processed series. The data series will first be divided into equal segments lasting 5 s, and the mean of each segment will be recorded for subsequent analysis.

b) Data analysis

We will calculate the mean SCL and HR values recorded for 4 min before the start of the negative mood induction procedure. These will be compared with the maximum scores obtained in the experimental phases (negative mood induction—viewing film clip—and emotional recovery using images). Independently for SCL and HR variables, repeated-measures ANOVA and post hoc Bonferroni tests will be performed in a mixed design with group (low, medium and high personal relevance groups) as between-subjects variable and time (pre-test—Time 1; post-negative emotional induction—Time 2; and postpositive emotional induction—Time 3) as within-subject variables.

##### EEG signal

- Data processing

The EEG recordings will be analysed off-line using custom software written in MATLAB. This custom software will be based on EEGLAB tools [98]. EEG functions will be used to clean the EEG-data. The EEG data will be segmented based on the event types. Fast Fourier transformation will be applied to an EEG epoch consisting of the time windows of 30 s each after the onset of the stimulus (film and images). Fourier transformed signals will then be averaged across event types. For further analysis, left frontal (F3, F7, FC5) and right frontal (F4, F8, FC6) electrode pools will be formed by averaging the frequency distributions of these signals. The mean magnitude of delta (0.5–4 Hz), theta (4.5–7.5 Hz), alpha (8–12.5 Hz), beta (13–30 Hz) and gamma (30.5–60 Hz) frequency band activities will be calculated for each participant during each event type.

b) Data analysis

A repeated-measures ANOVA will be performed to examine the possible effects of the intervention group (between-subjects variable) and time (within-subject variable) on the frequency band oscillations from frontal electrodes and on laterality (left vs right) by also examining the frequency band oscillations.

### Interim analyses {21b}

Not applicable. Given the characteristics of the study, potentially serious outcomes are not expected in the trial. For this reason, it is not necessary to develop guidelines to finish prematurely the trial due to adverse effects.

### Methods for additional analyses (e.g. subgroup analyses) {20b}

Not applicable. The allocation of the participants to the experimental groups is random, so it is expected that there are no differences between groups in relation to variables such as gender or educational level. In this sense, and taking into account the characteristics of the trial, it is not expected to have the need to perform subgroup analyses.

### Methods in analysis to handle protocol non-adherence and any statistical methods to handle missing data {20c}

Missing data (essentially, unanswered items from the screening measures) will be handled using a multiple imputation approach. This approach is based on the creation of a set of imputations for the respective variables with missing data. To this end, we will use a set of repeated imputations created by predictive models based on the majority of participants with complete data. After the imputations are completed, all of the data (complete and imputed) will be combined and the analysis performed for each imputed and completed dataset. Rubin’s method of multiple imputation will be used to estimate effects. We propose to use 100 datasets.

### Plans to give access to the full protocol, participant level-data and statistical code {31c}

No later than 2.5 years after completion of the data collection, we will deliver a completely deidentified data set to an appropriate data archive for sharing purposes.

## Oversight and monitoring

### Composition of the coordinating centre and trial steering committee {5d}

Not applicable. Given the characteristics of the study, there is no need for a data monitoring committee.

### Composition of the data monitoring committee, its role and reporting structure {21a}

Not applicable. This trial shows no minimal risks, and it is short duration. For these reasons, there is no need for a data monitoring committee.

### Adverse event reporting and harms {22}

Not applicable. It is not expected that the experimental intervention to produce negative effects. However, if after the phase of negative emotional induction, the intervention aimed at their emotional recovery was not effective, the psychologist responsible for the session, using the appropriate psychological techniques, will be responsible for the individual having recovered the emotional state previous to the start of the experimental session.

### Frequency and plans for auditing trial conduct {23}

Periodic meetings of all investigators will be established every 15 days to review processes related to (1) participant enrolment, consent, eligibility and allocation to study groups and (2) completeness, accuracy and timeliness of data collection.

### Plans for communicating important protocol amendments to relevant parties (e.g. trial participants, ethical committees) {25}

Any modification to the protocol that might influence how the study is conducted, including changes to the study objectives, study design, population characteristics, samples sizes or study procedures, will require a formal amendment to the protocol. Such amendments will be approved by the Clinical Research Ethics Committee of the Castilla-La Mancha Health Service prior to implementation.

### Dissemination plans {31a}

The primary outcome papers of the study will present outcome data about the effectiveness in emotion regulation of different levels of personal relevance of pictures as autobiographical memory cues to induce positive emotions. Additionally, we may on occasions be asked to contribute papers to workshops, symposia, congresses, etc.

The study is expected to end within the planned target of 2 years after completing data collection. We intend to reduce to the minimum the interval between the completion of data collection and the release of the study results. We expect to take about 4 to 5 months to elaborate the paper presenting the final results for an appropriate journal.

The study results will be released to the general psychology community.

## Discussion

The World Health Organization estimates that in 2050 there will be two billion people aged 65 years or older [99]. Population ageing, especially in developed countries, is an enormously important issue and is a challenge that heightens the need to create effective interventions to improve the mental health and quality of life of older persons. These predicted figures for population ageing also suggest there will be greater demand for effective alternative preventive treatments for depression, taking into account its prevalence in older adults [100, 101].

Emotion regulation is a key factor in adaptive functioning throughout life, being associated with cognitive, physical and social health [102]. This study protocol describes the design of a randomized controlled trial that aims to analyse how effective different types of autobiographical stimuli are in inducing positive emotions, analysing the variables that might enhance individuals’ emotion regulation for their use in the prevention of depressive symptoms.

The findings of this study will enhance knowledge of the functioning of positive mood induction procedures, which will help to better understand the processes involved in emotions in ageing and to assess the effect of ageing on emotional response and regulation and thus, also on older adults’ well-being. In addition, the results will lay the foundations for the development of future interventions designed to facilitate emotion regulation in both young and older adults, but above all in the latter, adapting the different techniques to the age group to ensure they are as effective as possible. In this sense, the knowledge derived from this study might allow individuals to use preventive techniques in their daily life and in their own homes, and thus the applicability of the findings will be considerable.

## Trial status

The original protocol was finished on December 20, 2019, which is the version to be used in this study.

Participant recruitment is expected to begin in October 2020 and continue for 2 months. Nevertheless, if the minimum number of participants in each experimental group is not reached in early December, the recruitment period will be extended until the required minimum is reached.

## Supplementary information

**Additional file 1.** Participant informed consent.

**Additional file 2.** Sociodemographic questionnaire.

## Abbreviations

ACC: Acceleration
ALUEX: Alumni Association of the University of Experience [*Asociación de Alumnos de la Universidad de la Experiencia*]
BCI: Brain Computer Interface
BVP: Blood volume pressure
EDA: Electrodermal activity
EEG: Electroencephalogram
FIR: Finite impulse response
GSR: Galvanic skin response
HR: Heart rate
HRV: Heart rate variability
HZ: Hertz
IAPS: International Affective Picture System
IBI: Interbeat interval
IBIs: Interbeat intervals
PANAS: Positive and Negative Affect Scale
PROMIS: Patient-Reported Outcomes Measurement Information System
SAM: Self-Assessment Manikin
SC: Signal
SCL: Skin conductance level
SCR: Skin conductance response
PNS: Parasympathetic nervous system
SNS: Sympathetic nervous system
SPSS: Statistical Package for the Social Sciences
TMP: Temperature
TYM: Test Your Memory
