# Supplementary material for: Effectiveness of the level of personal relevance of visual autobiographical stimuli in the induction of positive emotions in young and older adults: pilot study protocol for a randomized controlled trial
Source: Trials. 2020 Jul 20;21:663. doi: 10.1186/s13063-020-04596-5 (PMC7370414; doi:10.1186/s13063-020-04596-5)
Supplement: Supplementary file 1 — Additional file 1. Participant informed consent. [file 13063_2020_4596_MOESM1_ESM.docx]

**Additional file 1. PARTICIPANT INFORMED CONSENT.**

**INFORMATION SHEET AND CONSENT FORM FOR POTENTIAL PARTICIPANTS**

**Project title**

Intelligent environments controlled by physiological, perceptual and behavioral responses to emotional experiences.

**Study aim**

You are being invited to take part in a study whose aim is to broaden our knowledge of memory in relation to images.

**Study procedure**

The study requires collecting information from a group of adults. If you decide to participate, you will be asked to complete a series of questionnaires that will be presented in this session. If you are selected, you will then be asked to take part in a second session, in which an experiment related to images will be conducted.

**Risks and inconveniences**

This study involves no risk to your health or mood state, nor does it entail taking any medication.

**Voluntary nature**

Your participation in this study is entirely voluntary and you may withdraw at any time you wish.

**Confidentiality**

All the data collected on your participation in this study are considered confidential, and your participation will remain anonymous. The treatment of personal data in this study is regulated by Organic Law 15/1999 of 13 December, and by Royal Decree 1720/2007 of 21 December, which approves the regulation implementing said Organic Law (BOE number 17 of 19 January 2008). The consent for the processing and transfer of your personal data is always revocable. You may exercise your right to access, modify and remove any data by contacting the research team.

**Obtaining further information**

If you have any doubts about the study or your participation in it, you can speak to the research team at the Department of Psychology. Please contact Dolores Fernández Pérez at 96759920 or by email at Dolores.fernandez@uclm.es

I (name and surname) __________________________________________ have read and understood information about the project as provided in the information sheet.

I have been given the opportunity to ask questions about the study

I have been given sufficient information about the study.

I have spoken to (name of person in charge) ___________________________________

I understand that my participation is voluntary.

I understand that I can withdraw:

1º Whenever I wish.

2º Without the need to give any reasons.

3º Without any consequences.

I freely give my consent to participate in the study.

Date:

Participant’s signature:

Contact number: _____________________________________________________

If selected for the second session of the study, you will be notified at the phone number you provide.

As the person responsible for administering the booklet, I have fully explained the details of the study to the participant named above and I declare myself responsible for the veracity of the information recorded in this booklet.

Date:

Name and signature:

Note: A copy of this document has been given to the participant.

*For cases in which the participant is unable to read the document (cross out as appropriate).
