# Supplementary material for: Effectiveness of the level of personal relevance of visual autobiographical stimuli in the induction of positive emotions in young and older adults: pilot study protocol for a randomized controlled trial
Source: Trials. 2020 Jul 20;21:663. doi: 10.1186/s13063-020-04596-5 (PMC7370414; doi:10.1186/s13063-020-04596-5)
Supplement: Supplementary file 2 — Additional file 2. Sociodemographic questionnaire. [file 13063_2020_4596_MOESM2_ESM.docx]

**APPENDIX 2. SOCIODEMOGRAPHIC QUESTIONNAIRE**

| CODE (to be completed by the person responsible) | | | | | | | | |  |  | | |  | | | | |  |
| --- | --- | --- | --- | --- | --- | --- | --- | --- | --- | --- | --- | --- | --- | --- | --- | --- | --- | --- |
|  | | | | | | | | |  |  | | |  | | | | |  |
| DATE OF ASSESSMENT | | | | | | | | |  |  | | |  | | | | |  |
| DATE OF BIRTH | | | | | | | | |  |  | | |  | | | | |  |
| GENDER | | | |  | | | | | |  | | |  | | | | |  |
|  | Female | | | | | | | | | |  | |  | | | | |  |
|  | Male | | | | | | | | | |  | |  | | | | |  |
| EDUCATION | | | | | | |  | | | | | | | | |  |  |  |
|  | | | |  | | | | | | **YOU** | | | **YOUR PARTNER** | | | | |  |
| 1 | | Cannot read or write | | | | | | | |  | | |  | | | | |  |
| 2 | | No completed studies | | | | | | | |  | | |  | | | | |  |
| 3 | | Primary education to 5^th^ grade | | | | | | | |  | | |  | | | | |  |
| 4 | | Primary education to 8^th^ grade, High School, Compulsory Secondary Education, etc. | | | | | | | |  | | |  | | | | |  |
| 5 | | *BUP, COU*, Vocational Training, etc. | | | | | | | |  | | |  | | | | |  |
| 6 | | University degree | | | | | | | |  | | |  | | | | |  |
|  | |  | | | | | | | |  | | |  | | | | |  |
| CURRENT EMPLOYMENT SITUATION | | | | | | | |  | | | |  | |  | | | | |
|  | | | |  | | | | | | **YOU** | | | **YOUR PARTNER** | | | | |  |
| 1 | | Homemaker | | | | | | | |  | | |  | | | | |  |
| 2 | | Student | | | | | | | |  | | |  | | | | |  |
| 3 | | Unemployed | | | | | | | |  | | |  | | | | |  |
| 4 | | Employed | | | | | | | |  | | |  | | | | |  |
| 5 | | Self-employed or company owner | | | | | | | |  | | |  | | | | |  |
| 6 | | Retired or pensioner | | | | | | | |  | | |  | | | | |  |
|  | |  | | | | | | | |  | | |  | | | | |  |
| MARK WHICH OF THE FOLLOWING CURRENTLY IS, OR WAS UNTIL RETIREMENT, YOUR JOB ROLE AND THAT OF YOUR PARTNER | | | | | | | | | | | | | | |  |  |  |  |
|  | | | |  | | | | | | **YOU** | | | **YOUR PARTNER** | | | | |  |
| A1 | | | Self-employed without employees. | | | | | | |  | | |  | | | | |  |
| A2 | | | Self-employed with employees (less than ten). | | | | | | |  | | |  | | | | |  |
| A3 | | | Self-employed with employees (more than ten). | | | | | | |  | | |  | | | | |  |
| B1 | | | Manager in a company with less than ten employees. | | | | | | |  | | |  | | | | |  |
| B2 | | | Manager in a company with more than ten employees | | | | | | |  | | |  | | | | |  |
| B3 | | | Team leader or supervisor | | | | | | |  | | |  | | | | |  |
| B4 | | | Other type of employment without responsibility for staff. | | | | | | |  | | |  | | | | |  |
|  | | |  | | | | | | |  | | |  | | | | |  |
| CIVIL STATUS | | | | |  | | | | |  | | |  | | | | |  |
|  | | | | |  | | | | | **YOU** | | | | | | |  |  |
| 1 | | Single | | | | | | | |  | | |  | | | | |  |
| 2 | | Married | | | | | | | |  | | |  | | | | |  |
| 3 | | Divorced | | | | | | | |  | | |  | | | | |  |
| 4 | | Widowed | | | | | | | |  | | |  | | | | |  |
|  | |  | | | | | | | |  | | |  | | | | |  |
| RELATIONSHIP STATUS | | | | | |  | | | |  | | |  | | | | |  |
|  | | I have a partner | | | | | | | |  | | |  | | | | |  |
|  | | I have no partner | | | | | | | |  | | |  | | | | |  |

| INDICATE IF YOU HAVE ANY OF THESE HEALTH PROBLEMS | | | | |  | | |  | | |  | | |
| --- | --- | --- | --- | --- | --- | --- | --- | --- | --- | --- | --- | --- | --- |
|  | | | |  | | | | | **YES** | | | **NO** | |
|  | | High blood pressure | | | | | | |  | | |  | |
|  | | Diabetes | | | | | | |  | | |  | |
|  | | Arthritis and/or osteoarthritis | | | | | | |  | | |  | |
|  | | Osteoporosis | | | | | | |  | | |  | |
|  | | Hearing problems | | | | | | |  | | |  | |
|  | | Sight problems | | | | | | |  | | |  | |
|  | | Depression | | | | | | |  | | |  | |
|  | | Anxiety | | | | | | |  | | |  | |
|  | | Other health problems. Please specify: | | | | | | |  | | |  | |
| DO YOU TAKE REGULAR MEDICATION FOR ANY OF THE FOLLOWING HEALTH PROBLEMS? | | | | | | |  | |  | | |  | |
|  | | | | | | |  | | **YES** | | | **NO** | |
|  | | High blood pressure | | | | | | |  | | |  | |
|  | | Diabetes | | | | | | |  | | |  | |
|  | | Arthritis and/or osteoarthritis | | | | | | |  | | |  | |
|  | | Osteoporosis | | | | | | |  | | |  | |
|  | | Hearing problems | | | | | | |  | | |  | |
|  | | Sight problems | | | | | | |  | | |  | |
|  | | Depression | | | | | | |  | | |  | |
|  | | Anxiety | | | | | | |  | | |  | |
|  | | |  | | | | | |  | | |  | |
| HOW MANY **DIFFERENT** DRUGS DO YOU REGULARLY TAKE EACH DAY?  (e.g., 3 aspirins a day count as 1 drug; 1 aspirin and 1 paracetamol a day count as 2 drugs). | | | | | | |  | |  | | |  | |
|  | | | |  | | | | |  | | |  | |
| HOW DO YOU CONSIDER YOUR STATE OF HEALTH? | | | | | | |  | |  | | |  | |
| 1 | Very bad | | | | | | | |  | | |  | |
| 2 | Bad | | | | | | | |  | | |  | |
| 3 | Good | | | | | | | |  | | |  | |
| 4 | Very good | | | | | | | |  | | |  | |
|  | | |  | | | | | |  | | |  | |
| HOW DO YOU CONSIDER YOUR HEARING TO BE? | | | | | |  | | | |  | | |  |
| 1 | Very bad | | | | | | | |  | | |  | |
| 2 | Bad | | | | | | | |  | | |  | |
| 3 | Good | | | | | | | |  | | |  | |
| 4 | Very good | | | | | | | |  | | |  | |
